# The low prevalence rate of vitamin E deficiency in urban adults of Wuhan from central China: findings from a single-center, cross-sectional study

**DOI:** 10.1186/s40001-023-01103-9

**Published:** 2023-03-30

**Authors:** Ying Shen, Ke Liu, Xia Luo, Liming Cheng

**Affiliations:** Department of Laboratory Medicine, Tongji Hospital, Tongji Medical College, Huazhong University of Science and Technology, Wuhan, 430030 People’s Republic of China

**Keywords:** Vitamin E, Vitamin E deficiency, Urban adults, Wuhan, China

## Abstract

**Background:**

Vitamin E is an essential nutrient in human body famous for its antioxidant and non-antioxidant functions. However, little is known about vitamin E deficiency status in urban adults of Wuhan from central China. Our aim is to describe the distribution of both circulating and lipid-adjusted serum vitamin E concentration in urban adults of Wuhan.

**Methods:**

We hypothesized that the prevalence rate of vitamin E deficiency would be low in Wuhan in consideration of the Chinese food composition. A cross-sectional study with 846 adults was performed in a single-center. Concentrations of vitamin E were measured by liquid chromatography coupled with tandem mass spectrometry (LC–MS/MS).

**Results:**

The median (interquartile range, IQR) of serum vitamin E concentration was 27.40 (22.89–33.20) μmol/L while that of serum vitamin E concentration adjusted by total cholesterol or the sum of cholesterol (TC) and triglyceride (TG) (the sum of cholesterol and triglyceride, TLs) were 6.20 (5.30–7.48) and 4.86 (4.10–5.65) mmol/mol, respectively. No significant difference of the circulating and TC-adjusted vitamin E concentration was found between male and female except for vitamin E/TLs. However, concentrations of vitamin E increased significantly (*r* = 0.137, *P* < 0.001) with age, but lipid-adjusted concentrations of vitamin E did not. On analysis of risk factors, the subjects characterized by hypercholesterolemia are more likely to exhibit higher circulating but lower lipid-adjusted vitamin E level due to adequacy of the serum carriers for delivery of vitamin E. Only 0.47% of the population were below 12 μmol/L of vitamin E defined as functional deficiency.

**Conclusion:**

The prevalence rate of vitamin E deficiency in urban adults of Wuhan is low, which is important and useful to clinicians for clinical decision-making in public health practice.

**Supplementary Information:**

The online version contains supplementary material available at 10.1186/s40001-023-01103-9.

## Background

Vitamin E is an important fat-soluble nutrient in the maintenance of health famous for its antioxidant functions, beneficial for a variety of disorders including cancer, heart disease and even Parkinson’s disease [1–4]. Meanwhile vitamin E also appears to have a variety of roles depending on its non-antioxidant properties, such as modulation of monocyte function, inhibition of platelet aggregation, inhibition of smooth muscle cell proliferation and modulation of gene expression [5]. Up to now, the extensive implications of vitamin E deficiency are increasing evident, especially in developing countries whose risk for deficiency is higher due to limited intake of the vitamins from food sources and greater oxidative stressors [6]. As a result, we initiate the present study to evaluate vitamin E status in our city from China. As well known, vitamin E covers a group of eight compounds (α-, β-, γ-, δ-tocopherol, and α-, β-, γ-, δ-tocotrienol) which differ in their methyl substitution and saturation. Among them, the predominant form in the human body is α-tocopherol which demonstrates the highest vitamin E activity, comprising over 90% of vitamin E [7–9]. Consequently, vitamin E status is always assessed by serum α-tocopherol concentration which provides the mostly used and direct way [10]. It is reported that vitamin E circulating in blood is transported by lipoproteins, and vitamin E partitioning out of the cellular membrane compartment would increase with the elevation of serum lipid concentrations [11]. Consequently, vitamin E deficiency may be underestimated without consideration of the lipid concentration in the case that serum lipid concentrations pathologically elevated, while may be overestimated under the situation that serum lipid concentrations are low. Many researches thus suggest that the correction of vitamin E for lipid concentrations is preferable to assess adequacy [12]. Oftentimes, serum α-tocopherol concentration adjusted for serum total cholesterol (TC) or the sum of serum levels of cholesterol and triglyceride (TG) (the sum of cholesterol and triglyceride, TLs) is considered as a reliable indicator in identifying vitamin E deficiency [13, 14]. Nonetheless, the vitamin E status of people in Wuhan from central China is less-known, and an understanding of the vitamin E distribution in our city should be useful to clinicians for clinical decision-making in public health practice.

In this study, both the circulating serum a-tocopherol concentrations and serum a-tocopherol concentrations adjusted for lipids have been used to assess the nutritional status of vitamin E of urban adults in Wuhan. Wuhan is located on the banks of the Yangtze River in central China, and the food consumption has dominated by traditional Chinese food characterized by grains and vegetables, with increasing intake of red meat, fruits, nuts, eggs, milk, river fish and plant oil, most of which are rich in vitamin E [15, 16]. Thus, we hypothesized that the likelihood of vitamin E deficiency would be low in Wuhan in consideration of the Chinese food composition.

## Methods

### Study design and subjects

A single-center, cross-sectional study was performed. All subjects, residing in Wuhan, were enrolled from those who underwent physical examination program in 2019 at Tongji Hospital of Tongji Medical College of Huazhong University of Science and Technology (HUST) which is a large comprehensive hospital in Wuhan with abundant patients from local residents. The basic demographic characteristics of the participants including sex, age, BMI, blood pressure and long-term residence place were collected through medical records or face-to-face interview. This work was approved by the ethics committee of Tongji Hospital, Tongji Medical College, Huazhong University of Science and Technology (IRB Approval Number: TJ-IRB20210807).

The sample size was calculated using the formula *N* = [*Z*_1-α/2_]^2^ × *P* (1-* P*)/*d*^2^ [17]. Where *N* is the sample size, *Z*_1-α/2_ (1.96) is the certainty wanted expressed in the percentage point of normal distribution corresponding to the 2-sided level of significant (*α* = 0.05); *P* (13%) is the global prevalence rate of vitamin E deficiency [18]; *d* (3%) is the allowable error. Therefore, *N* = [(1.96)^2^ × 0.13 × (1–0.13)]/(0.03)^2^ = 483. A non-response rate of 40% was added, giving a total sample of 805. In view of incomplete demographic data, a total of 850 samples were ready to be included. Finally, 846 samples were selected in real word according to the actual situations, including 471 males and 375 female aged 18 to 93 years (median age 47 years).

Overnight fasting blood samples were obtained by venipuncture. Sera were obtained by centrifugation of coagulated blood samples at 3000 rpm for 5 min at room temperature. These sera were frozen and stored at − 80 °C until analysis.

### Measures

The serum α-tocopherol concentrations were determined by liquid chromatography coupled with tandem mass spectrometry (LC–MS/MS) on a ABsciex Qtrap 5500 coupled to an Exion LC system (Applied Biosystems, Foster City, CA, USA) with an electrospray ionization source in positive mode, and the testing kit was gotten from Beijing Health biotech Co. Ltd. (Beijing, China). This method was well validated with linearity, precision, accuracy, analytical sensitivity and matrix effect as demonstrated in Additional file 1: Table S1. The sera were processed as follows: 0.1 ml of serum was mixed with 0.1 ml of the internal standard (α-tocopherol-d6, Sigma-Aldrich) solution in a 1.5-ml centrifuge tube; 0.6 ml of hexane was then added and mixed thoroughly for 3 min using a vortex mixer. The tube was then closed and centrifuged for 10 min at 14680 rpm. The upper hexane extracts were evaporated by nitrogen and reconstituted in acetonitrile for LC–MS/MS analysis. A symmetry C18 column (100 × 2.1 mm, 3.5 µm, Waters, USA) was used for separation. The mobile phase was consisted of solvent A (water with 0.1% formic acid) and solvent B (2 mM ammonium acetate with 0.1% formic acid in methanol). The flow rate was 0.7 ml/min and column temperature was 60 °C. The concentrations of serum lipids, including TC, TG, high-density lipoprotein cholesterol (HDLC) and low-density lipoprotein cholesterol (LDLC), were measured on a Cobas 8000 system (Roche, Diagnostics, Germany). During the analysis, samples were protected from light and those showing signs of hemolysis were discarded.

In this study, the criterion for hypercholesterolemia is defined as TC ≥ 5.2 mmol/L [19]. The normal blood pressure recommended by WHO is < 140/90 mmHg [20]. The standard weight status categories associated with BMI ranges for adults are: BMI below 18.5 is associated with ‘underweight’ weight status; BMI 18.5–24.9 is associated with ‘normal’ weight status; BMI 25.0–29.9 is associated with ‘overweight’ weight status; BMI 30.0 and above is associated with ‘obese’ weight status [21]. The vitamin E status categories for healthy adults are classified as follows [18, 22]: vitamin E serum concentrations ≤ 12 μmol/L is considered as functional deficiency; between 13 and 29 µmol/L is considered as suboptimal status; ≥ 30 μmol/L is considered as desirable status. The prevalence rate of vitamin E deficiency according to its status categories defined for healthy adults, and its comparison with other countries have been investigated.

### Statistical analysis

We present the distribution of concentrations of vitamin E and the ratios of concentrations of vitamin E to serum lipids for all participants. All data were expressed as medians and interquartile ranges (IQRs). Data normality was analyzed using the Kolmogorov–Smirnov test. Student’s *t* test and analysis of variance (ANOVA) based on normally distributed data, or Mann–Whitney *U* test and Kruskal–Wallis test based on non-normally distributed data were applied to compare the means of serum concentrations of α-tocopherol across genders, age groups and strata. Pearson’s Chi-square test or Fisher’s exact test was performed to analyze the categorical data. Spearman’s correlation test was used to determine the strength of correlation between variables, such as vitamin E, age, BMI, blood pressure, TC and TG.

A multivariate logistic regression was performed to assess odds ratios (ORs) regarding Vitamin E-associated factors. Variable with a global value *P* < 0.10 in the univariate analysis were entered into multivariate analyses. A *P*-value below 0.05 was considered statistically significant. Analyses were done using SPSS (version 20.0; SPSS, Isnc., Chicago, Ill, USA). The Correlation analysis heatmap was plotted by R language.

## Results

### Profile of the study population

The basic characteristics of the study population of 846 subjects aged 18 to 93 years are demonstrated in Table 1. The median (interquartile range, IQR) of age and body mass index (BMI) were 47 (36–56) and 24.1 (21.9–26.1), respectively. The median (IQR) of blood pressures were found within normal range: 124 (113–137) mmHg for systolic blood pressure (SBP) and 76 (69–85) mmHg for diastolic blood pressure (DBP). All the subjects had measurements for concentrations of TC, HDLC, and LDLC, TG and α-tocopherol. Both levels of α-tocopherol unadjusted and adjusted for lipids were presented.

**Table 1:** Serum concentrations of α-tocopherol per sex in adults (*n* = 846)^a^

	Total	Male	Female	*P*
*N* (%)	846	471 (55.67%)	375 (44.33%)	
Age (years)	47 (36–56)	47 (36–56)	47 (34–56)	0.422
BMI (kg/m^2^)	24.1 (21.9–26.1)	25.1 (23.3–27.0)	22.6 (20.7–24.8)	< 0.001
SBP (mm Hg)	124 (113–137)	127 (116–140)	119 (108–132)	< 0.001
DBP(mm Hg)	76 (69–85)	80 (73–87)	72 (65–81)	< 0.001
TC (mmol/L)	4.41 (3.89–4.99)	4.41 (3.92–4.99)	4.40 (3.88–5.00)	0.788
TG (mmol/L)	1.16 (0.82–1.70)	1.39 (1.00–1.96)	0.97 (0.68–1.40)	< 0.001
HDLC (mmol/L)	1.25 (1.07–1.49)	1.15 (1.00–1.29)	1.43 (1.25–1.65)	< 0.001
LDLC (mmol/L)	2.77 (2.29–3.32)	2.84 (2.38–3.40)	2.69 (2.23–3.26)	0.030
TLs (mmol/L)	5.65 (4.97–6.64)	5.88 (5.14–6.86)	5.43 (4.81–6.31)	< 0.001
Unadjusted				
Vitamin E (μmol/L)	27.40 (22.89–33.20)	27.63 (22.96–32.97)	27.16 (22.82–33.20)	0.683
Adjusted for				
TC (mmol/mol)	6.20 (5.30–7.48)	6.28 (5.36–7.55)	6.16 (5.24–7.27)	0.369
TLs (mmol/mol)	4.86 (4.10–5.65)	4.70 (3.97–5.53)	4.98 (4.31–5.86)	< 0.001

^a^Data are presented with median (IQR) for continuous variables

*SBP* systolic blood pressure, *DBP* diastolic blood pressure, *TLs* total lipids (cholesterol + triglyceride)

### The association of vitamin E level with the physiological conditions

**Fig. 1:**
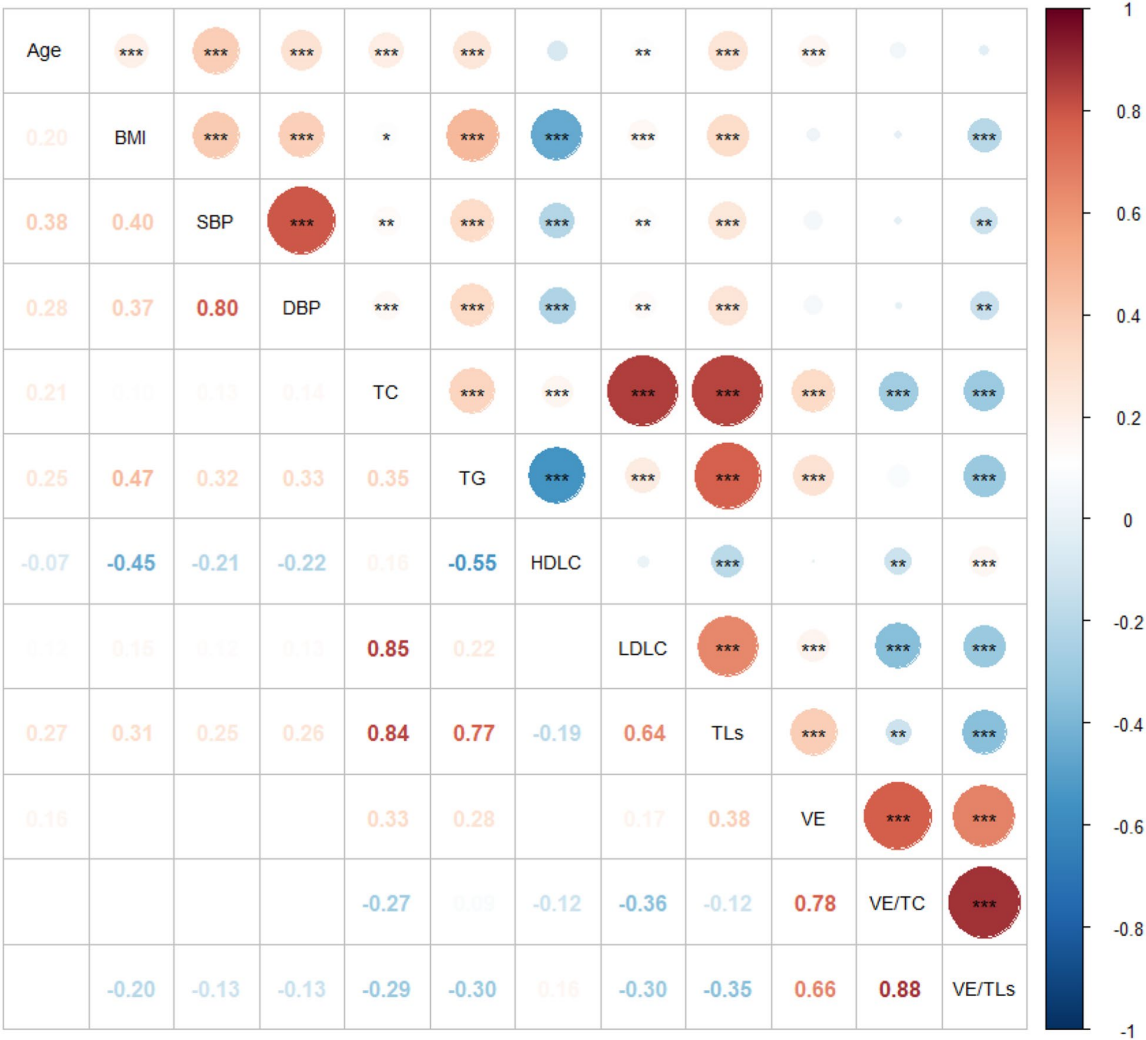
Spearman’s correlation coefficients between parameters in adults (*n* = 846)^1^. 1***P < 0.001, **P < 0.01, *P < 0.05. The color bar on the right side represents Spearman’s correlation coefficients in the range of − 1.0 (blue color)-1.0 (red color). The redder the color and the larger the circle, the stronger the positive correlation; the bluer the color and the larger the circle, the stronger the negative correlation. *BMI* body mass index, *SBP* systolic blood pressure, *DBP* diastolic blood pressure, *TC* total cholesterol, *TG* triglyceride, *HDLC* high-density lipoprotein cholesterol, *LDLC* low-density lipoprotein cholesterol, *TLs* total lipids (the sum of cholesterol and triglyceride), *VE* vitamin E, *VE/TC* vitamin E/total cholesterol, *VE/TLs* vitamin E/total lipids

The association of vitamin E level with sex and age was investigated. Analysis of the data, noted as a function of sex (Table 1), shown that men had significant higher BMI (*P* < 0.001), SBP (*P* < 0.001), DBP (*P* < 0.001), TG (*P* < 0.001) and LDLC (*P* = 0.030) than women, and inversely had lower HDLC (*P* < 0.001) and vitamin E/TLs (*P* < 0.001) than women with no difference found in age, TC, vitamin E and vitamin E/TC. Likewise, the median levels of all indicators in Table 2 varied significantly with age except for vitamin E/TC (*P* = 0.573) and vitamin E/TLs (*P* = 0.131). This was also confirmed by the positive correlation between age and BMI (*r* = 0.183, *P* < 0.001), SBP (*r* = 0.409, *P* < 0.001), DBP (*r* = 0.214, *P* < 0.001), TC (*r* = 0.152, *P* < 0.001), TG (*r* = 0.126, *P* < 0.001), LDLC (*r* = 0.073, *P* < 0.05), TLs (*r* = 0.168, *P* < 0.001), vitamin E (*r* = 0.137, *P* < 0.001) (Fig. 1). Nonetheless, only HDLC, vitamin E/TC and vitamin E/TLs did not vary in trend, resulting in low Spearman correlation coefficients or insignificant associations between different age subgroups.

**Table 2:** Serum concentrations of α-tocopherol per age group in adults (*n* = 846)

	18–29 years	30–39 years	40–49 years	50–59 years	60–69 years	≥ 70 years	*P*
*N* (%)	107 (12.65%)	174 (20.57%)	201 (23.76%)	206 (24.35%)	79 (9.34%)	79 (9.34%)	
Age (years)	26.00 (24.00–28.00)	35.00 (32.00–37.00)	45.00 (42.00–47.00)	54.00 (52.00–56.00)	65.00 (62.00–67.00)	78.00 (73.00–84.00)	< 0.001
BMI (kg/m^2^)	21.20 (19.10–24.70)	23.80 (21.20–26.10)	24.50 (22.30–26.05)	24.65 (22.68–26.9)	24.20 (22.30–26.00)	24.70 (22.30–27.30)	< 0.001
SBP (mm Hg)	116 (104–129)	118.5 (109.0–130.0)	119 (111–130)	127.5 (117–141)	129 (120–144)	147 (133–158)	< 0.001
DBP (mm Hg)	71 (64–78)	74 (67–80)	76 (67–83.5)	82 (71.75–89)	79 (72–87)	82 (74–86)	< 0.001
TC (mmol/L)	4.03 (3.60–4.49)	4.20 (3.78–4.77)	4.48 (3.96–5.03)	4.72 (4.22–5.20)	4.61 (4.05–5.22)	4.38 (3.78–4.94)	< 0.001
TG (mmol/L)	0.82 (0.61–1.19)	1.12 (0.80–1.62)	1.13 (0.82–1.68)	1.40 (0.92–2.06)	1.28 (0.99–1.77)	1.44 (1.03–1.83)	< 0.001
HDLC (mmol/L)	1.36 (1.20–1.57)	1.20 (1.05–1.43)	1.26 (1.06–1.50)	1.24 (1.03–1.50)	1.26 (1.10–1.49)	1.22 (1.01–1.42)	0.009
LDLC (mmol/L)	2.45 (2.11–3.02)	2.66 (2.23–3.23)	2.83 (2.33–3.32)	3.00 (2.60–3.48)	2.85 (2.33–3.57)	2.60 (1.99–3.33)	< 0.001
TLs (mmol/L)	4.95 (4.30–5.70)	5.33 (4.73–6.24)	5.65 (5.07–6.66)	6.13 (5.38–7.11)	5.84 (5.36–6.85)	5.93 (4.93–6.69)	< 0.001
Unadjusted							
Vitamin E (μmol/L)	24.84 (21.41–30.88)	25.88 (21.85–30.65)	27.63 (23.10–34.59)	28.32 (23.85–34.19)	28.32 (23.91–35.29)	28.32 (22.33–35.52)	< 0.001
Adjusted for							
TC (mmol/mol)	6.15 (5.42–7.25)	6.16 (5.34–7.13)	6.36 (5.34–7.41)	6.14 (5.21–7.44)	6.46 (5.22–7.62)	6.71 (5.35–8.21)	0.573
TLs (mmol/mol)	5.08 (4.31–6.03)	4.82 (4.18–5.49)	4.86 (4.10–5.72)	4.68 (3.94–5.39)	4.73 (4.10–5.72)	5.01 (4.02–6.26)	0.131

### The association of vitamin E level with risk factors

Many people suffer from risk factors as critical links to the development of diseases, such as TC level, blood pressure or BMI. Thus, the α-tocopherol levels on these risk factors were also analyzed. No difference was found in BMI and DBP as presented in Additional file 1: Table S2. However, the people with hypercholesterolemia have significantly older age (*P* < 0.001), higher SDP (*P* = 0.036), TC (*P* < 0.001), TG (*P* < 0.001), HDLC (*P* = 0.007), LDLC (*P* < 0.001), TLs (*P* < 0.001) and vitamin E levels (*P* < 0.001) while significantly lower vitamin E/TC (*P* < 0.001) and vitamin E/TLs levels(*P* < 0.001) (Table 3 and Additional file 1: Table S2). These parameters were further confirmed by Spearman’s correlation coefficients between TC and these indicators (Fig. 1). The people were divided into two groups. The population with high blood pressure exhibited older age (*P* < 0.001), higher BMI (*P* < 0.001), higher TC (*P* = 0.005), TG (*P* < 0.001) and TLs (*P* < 0.001) levels with inversely lower HDLC (*P* < 0.001) and vitamin E/TLs levels (*P* = 0.020). Nonetheless, no differences were found in LDLC (*P* = 0.058), vitamin E (*P* = 0.105) and vitamin E/TC (*P* = 0.560) levels, which were also evidenced by the insignificant correlation between SBP, DBP and vitamin E or vitamin E/TC except for LDLC (Table 3, Additional file 1: Table S3 and Fig. 1).

**Table 3:** The analysis of α-tocopherol levels on risk factors

	*N*	Male (*n* %)	Age	VE (μmol/L)	VE/TC (mmol/mol)	VE/TLs (mmol/mol)
TC level						
Hypercholesterolemia (total cholesterol ≥ 5.2 mmol/L)	155 (18.32%)	84 (54.19%)	51 (43–57)	32.5 (26.00–39.74)	5.65 (4.58–6.93)	4.36 (3.60–5.16)
Normal (total cholesterol < 5.2 mmol/L)	691 (81.68%)	387 (56.01%)	45 (34–56)	26.7 (22.45–31.58)	6.35 (5.46–7.55)	4.91 (4.22–5.73)
*P*		0.681	< 0.001	< 0.001	< 0.001	< 0.001
Blood pressure						
High blood pressure (≥ 140/90 mmHg)	210 (24.82%)	145 (69.05%)	56 (46–70)	28.32 (22.92–34.48)	6.36 (5.29–7.80)	4.72 (3.90–5.46)
Normal (< 140/90 mmHg)	636 (75.18%)	326 (51.25%)	44 (33–53)	27.16 (22.89–32.50)	6.16 (5.32–7.37)	4.90 (4.16–5.70)
*P*		< 0.001	< 0.001	0.105	0.560	0.020
BMI						
< 18.5 (under weight)	23 (2.72%)	4 (17.39%)	27 (21–29)	24.61 (21.41–28.32)	6.48 (5.42–8.12)	5.35 (4.51–6.13)
18.5–24.9 (normal weight)	488 (57.68%)	223 (45.70%)	47 (35–56)	27.40 (22.62–33.20	6.18 (5.37–7.42)	4.96 (4.22–5.87)
25.0–29.9 (over weight)	303 (35.82%)	222 (73.27%)	49 (40–57)	27.40 (23.45–33.20)	6.26 (5.26–7.61)	4.64 (3.88–5.45)
≥ 30.0 (obese)	32 (3.78%)	22 (68.75%)	41 (32–62)	28.32 (22.54–32.97)	6.26 (4.89–7.02)	4.48 (3.78–5.30)
*P*		< 0.001	< 0.001	0.128	0.576	0.094

In order to evaluate whether there were differences in vitamin E, lipid-adjusted vitamin E or other indicators between BMI subgroups, we could only perform Kruskal–Wallis test in consideration of abnormal distribution of theses variables. As demonstrated in Table 3, with the increase of age, the people were apt to be overweight or obese (the median of age was above 41 years old). The people in group with higher BMI exhibited significantly increased levels of blood pressure, TC, TG, LDLC and TLs (all *P* values below 0.008), but decreased HDLC concentration (*P* < 0.001). Nevertheless, there were no differences in vitamin E, vitamin E/TC and vitamin E/TLs (Table 3 and Additional file 1: Table S4). These results were in agreement with the Spearman’s correlation analysis between BMI and other indicators as revealed in Fig. 1 except for vitamin E/TLs which presented strengthened correlation with BMI.

### The distribution of vitamin E

The classification of subjects according to their α-tocopherol levels is presented in Table 4. In this study, 0.47% of the population were below 12 μmol/L (functional deficiency), 61.47% between 12 and 30 μmol/L (suboptimal concentration), and 38.06% above 30 μmol/L (desirable concentration). As a stratification by sex, the males and females exhibited similar distributions as the total did. As described above, a significant positive association was revealed between age and α-tocopherol. Therefore, as a stratification by age, the higher proportion of people above the desirable concentration was found in older age group with a maximal value of 45.57%, but the proportion was slightly gone down again in people above 70 years old.

**Table 4:** Classification of the subjects according to their serum concentrations of α-tocopherol in adults

	≤ 12 μmol/L (Deficient)	13–29 μmol/L (Suboptimal)	≥ 30 μmol/L (Desirable)
Total (*n* = 846)	4 (0.47%)	520 (61.47%)	322 (38.06%)
Sex			
Male (*n* = 471)	2 (0.42%)	289 (61.36%)	180 (38.22%)
Female (*n* = 375)	2 (0.53%)	231 (61.60%)	142 (37.87%)
Age			
18–29 (*n* = 107)	0	77 (71.96%)	30 (28.04%)
30–39 (*n* = 174)	0	123 (70.69%)	51 (29.31%)
40–49 (*n* = 201)	3 (1.49%)	117 (58.21%)	81 (40.30%)
50–59 (*n* = 206)	0	116 (56.31%)	90 (43.69%)
60–69 (*n* = 79)	0	43 (54.43%)	36 (45.57%)
≥ 70 (*n* = 79)	1 (1.27%)	44 (55.70%)	34 (43.03%)

Regarding that there are only four cases with vitamin E deficiency, subjects with insufficiency and deficiency (< 30 μmol/L) were grouped together to further assess factors associated with vitamin E status, which referred the subjects with vitamin E sufficiency (≥ 30 μmol/L) as a comparison group. The factors determining vitamin E insufficiency and deficiency derived from multivariate logistic regression analyses are shown in Table 5. The analysis confirmed the significant association between vitamin E status and age (OR: 0.986; 95% CI 0.976–0.996), and vitamin E status and TC level (OR: 0.305; 95% CI 0.212–0.439).

**Table 5:** Association between serum vitamin E status and factors

	Vitamin E^a^	*P*
	Insufficiency and deficiency, OR (95% CI)	
Participates, *n*	524	
Age	0.986 (0.976–0.996)	0.007
TC level		< 0.0001
Normal	1	
Hypercholesterolemia	0.305 (0.212–0.439)	
Blood pressure		0.838
Normal	1	
High blood pressure	0.964 (0.678–1.371)	

^a^Desirable vitamin E levels: α-tocopherol ≥ 30 μmol/L (comparison group)

## Discussion

As the diverse role of vitamin E, especially function as a potent antioxidant in the maintenance of health and prevention of disease, the extensive implications of its deficiency are increasingly evident. As usual, serum vitamin E should be transported to tissues for exerting their functions by using lipoproteins as the major carries [23]. In humans, vitamin E is mostly transported in LDL and HDL at similar proportions with less carried in VLDL and other lipoproteins. As a result, vitamin E homeostasis is intimately connected to lipoprotein metabolism in vivo. Assessment of vitamin E status not only depends on its concentration, but also on the concentrations of the circulating lipoproteins. In fact, vitamin E deficiency is rare in humans. When it occurs, it is a result of lipoprotein deficiencies or lipid malabsorption syndromes. It is important to identify patients who have real vitamin E deficiency or other abnormalities known to cause vitamin E deficiency [24]. Thus, concentration of vitamin E, both unadjusted and adjusted for cholesterol or total lipids, should be used to evaluate vitamin E adequacy [25]. This study confirmed that the prevalence rate of vitamin E deficiency is low in urban adults of Wuhan based on both unadjusted and lipid-adjusted vitamin E levels, which provided valuable information about the distribution of vitamin E in central China.

In this study, we found that the vitamin E level was not associated with sex as revealed in Table 1 (27.63 vs 27.16 μmol/L, *P* = 0.683), whereas the vitamin E level slightly increased with age in Table 2. The results were consistent with the previous report [26]. Meanwhile, the levels of BMI, SBP, DBP, TG and LDLC were significantly higher in men, mostly in consistent with Sun’s work [27]. However, an opposite direction of association in lipoprotein between men and women was found in Qi’s study that TC was significantly lower in men with no TG difference [28], which might be due to dietary intake or life habit in different regions. Likewise, it is well understood that aging process affect BMI, blood pressure and lipoprotein which are risk factors for cardiovascular diseases as Yao’s or Mendes’s report [29, 30].

In order to learn about the vitamin E level in these subjects with risk factors for cardiovascular diseases potentially imposing a great threat to human health, the subjects were divided into subgroups according to TC level, blood pressure and BMI. The transport of vitamin E is closely related to lipoprotein [24]. Therefore, hypercholesterolemia might affect the vitamin E level. As demonstrated in Fig. 1, the level of vitamin E is positively correlated with TC, TG, LDLC and TLs (*P* < 0.05), and much higher level of the vitamin E is found in subjects with hypercholesterolemia. In general, the participates who have increased blood lipid concentration also have increased serum vitamin E level, accompanied by evidently decreased lipid-adjusted vitamin E levels [25, 31]. This might be ascertained to the increased serum carriers for delivery of vitamin E in tissues [11]. Although TC level, blood pressure and BMI are highly correlated with each other as displayed in the Spearman analysis (Fig. 1) and stratification analysis (Additional file 1: Tables S2, S3, S4) [32], no significant difference of vitamin E, vitamin E/TC or vitamin E/TLs is found between subgroups based on blood pressure or BMI except that vitamin E/TLs is lower in subjects with hypertension. Obesity might be the most important factor associated with blood pressure, followed by hyperlipidemia [33], which contributes to the consistent result between blood pressure and BMI.

In this study, a considerable proportion of population presents suboptimal vitamin E status (61.47%), while a very small population exhibits vitamin E deficiency (0.47%). Both age and TC level are significantly correlated with vitamin E status, which are indirectly evidenced by associations of vitamin E concentrations and variables (Table 2 and Table 3). It is found that almost equal proportions of the men and women in distribution of vitamin E. However, the young adults (18–39 years old) exhibited lower proportion of desirable vitamin E level than that of older, which was not consistent with Oldewage-Theron’s study [34]. Perhaps, the older adults in urban China have paid more attention to vitamin/trace element supplements in daily life due to their enhanced awareness of health. However, poor food intake as well as a monotonous diet in the elderly above 70 years old made the prevalence of vitamin E deficiency suffered. In addition, the vitamin E level in different countries is summarized in Table 6 [18, 26, 34–38]. Compared to the results from other countries, the serum vitamin E level in our subjects is higher than that of most countries and in parallel with that of US, even above the global level. Given that the cut-off point of vitamin E is 12 μmol/L, the prevalence of vitamin E inadequacy for urban adults in Wuhan from central China is 0.47%, much lower than that of other countries. Meanwhile, if 11.6 μmol/L was adopted as the cut-off value [39], the deficiency rate was 0.24%. It is noted that there is a great variation in blood lipids across nations and vitamin E is transported in lipoprotein fraction in the blood which is determinant for its concentration. However, a few studies provide lipid-adjusted vitamin E, thus it is not clear that whether the prevalence defined is comparable in various countries. If vitamin E deficiency was expressed as < 2.5 mmol/mol total cholesterol or expressed as < 1.59 mmol/mmol (total cholesterol + triacylglycerols) which was too uncommon to report [36], the prevalence of deficiency was 0.47% or 0.35% in Wuhan, respectively. Inspiringly, no matter what methods were used to adjust or evaluate for vitamin E concentration, the subjects in this study were identified as being vitamin E sufficient in comparison with other countries or regions. The low prevalence of deficiency in present study might due to the fact that the Chinese diet depends on vegetable oils, meat, green leafy vegetables, cereals, wheat germ and egg yolk, which are known to be the principal dietary sources of vitamin E [40].

**Table 6:** Comparison of the vitamin E status between Wuhan in central China and other countries

Reference	Location	No	Age	Definition of deficiency	% Deficiency	Median or mean of vitamin E (μmol/L)	Median or mean of vitamin E/TC (mmol/mol)
This study, 2020	Wuhan, central China	846	18–93	Serum α-tocopherol ≤ 12.0 μmol/L (I) OR < 11.6 (II) OR serum α-tocopherol: cholesterol < 2.5 mmol/mol (III) OR α-tocopherol:(cholesterol + triacylglycerols) < 1.59 mmol/mol (IV)	0.47(I) 0.24(II) 0.47(III) 0.35(IV)	27.40	6.20
Péter et al., 2016 [18]	Global	132 studies	NA	Serum α-tocopherol ≤ 12.0 μmol/L	13	22.1	NA
Oldewage-Theron et al., 2009 [34]	Sharpeville, South Africa	235	60–93 yr	Serum α-tocopherol < 2.8 μmol/L (I) OR < 3.7 μmol/L (II)	20.9 (I) 16.2 (II)	4.8	NA
Assantachai et al.,2007 [35]	Thailand	2336	≥ 60 yr	Plasma α-tocopherol < 14 μmol/L	55.5	NA	NA
Ford et al.,2006 [36]	US	4087	≥ 20 yr	Serum α-tocopherol < 11.6 μmol/L	0.50	27.39	4.93
Obeid et al., 2006 [37]	Beirut, Lebanon	857	25–64 yr	Plasma α-tocopherol < 5.8 μmol/L (I) OR < 11.6 (II) OR plasma α-tocopherol: cholesterol < 2.5 μmol/mmol (III)	0.7 (I) 3.7 (II) 4.1 (III)	24.5	4.67
Gouado et al., 2005 [26]	Northern Cameroon	81	3–61 yr	Serum α-tocopherol < 5.8 μmol/L (I) OR < 11.6 μmol/L (II)	12.3 (I) 33.3 (II)	12.2	NA
Kang et al., 2004 [38]	Taiwan	1841	≥ 19 yr	Serum α-tocopherol < 11.6 μmol/L	7.2	20.0	NA

Certain limitations should be noted when interprets the result of this study. Firstly, the results are based on cross-sectional data and the sample size is small. Secondly, this a single-center study, and thus its explanatory power is limited. Thirdly, the information for dietary supplements is deficient to know about the relationship between food sources and the serum vitamin E level.

## Conclusion

The prevalence of vitamin E deficiency was lower in Wuhan from central China than other countries. The vitamin E was closely associated with lipids in comparison with BMI or blood pressure. We hope that the information in this study would prove useful to learn about vitamin E status of urban adults in China.

## Supplementary Information

**Additional file 1: Table S1** Method validation. **Table S2** Classification of the subjects according to their TC levels in adults (n=846). **Table S3** Classification of the subjects according to their blood pressures in adults (n=846). **Table S4** Classification of the subjects according to their BMI in adults (n=846).

## Data Availability

The data could be available if needed.

## Abbreviations

ANOVA: Analysis of variance
BMI: Body mass index
SBP: Systolic blood pressure
DBP: Diastolic blood pressure
IQRs: Interquartile ranges
LC–MS/MS: Liquid chromatography coupled with tandem mass spectrometry
LDLC: Low-density lipoprotein cholesterol
HDLC: High-density lipoprotein cholesterol
TC: Total cholesterol
TG: Triglyceride
TLs: Total lipids (the sum of cholesterol and triglyceride)

## References

[1] Lodge JK (2008). Mass spectrometry approaches for vitamin E research. Biochem Soc Trans.

[2] Zhang XH, Feng MH, Liu F, Qin L, Qu RJ, Li DL (2014). Subacute oral toxicity of BDE-15, CDE-15, and HODE-15 in ICR male mice: assessing effects on hepatic oxidative stress and metals status and ascertaining the protective role of vitamin E. Environ Sci Pollut Res.

[3] Manosso LM, Camargo A, Dafre AL, Rodrigues ALS (2020). Vitamin E for the management of major depressive disorder: possible role of the anti-inflammatory and antioxidant systems. Nutr Neurosci.

[4] Matsura T (2019). Protective effect of tocotrienol on in vitro and in vivo models of Parkinson’s disease. J Nutr Sci Vitaminol.

[5] Zingg JM, Azzi A (2004). Non-antioxidant activities of vitamin E. Curr Med Chem.

[6] Dror DK, Allen LH (2011). Vitamin E deficiency in developing countries. Food Nutr Bull.

[7] Jiang Q (2014). Natural forms of vitamin E: metabolism, antioxidant, and anti-inflammatory activities and their role in disease prevention and therapy. Free Radic Biol Med.

[8] Hall WL, Jeanes MY, Lodge JK (2005). Hyperlipidemic subjects have reduced uptake of newly absorbed vitamin E into their plasma lipoproteins, erythrocytes, platelets, and lymphocytes, as studied by deuterium-labeled alpha-tocopherol biokinetics. J Nutr.

[9] Jensen SK, Lauridsen C (2007). Alpha-tocopherol stereoisomers. Vitam Horm.

[10] Azzini E, Polito A, Fumagall A, Intorre F, Venneria E, Durazzo A (2011). Mediterranean diet effect: an Italian picture. Nutr J.

[11] Ford L, Farr J, Morris P, Berg J (2006). The value of measuring serum cholesterol-adjusted vitamin E in routine practice. Ann Clin Biochem.

[12] Traber MG (2014). Vitamin E inadequancy in humans causes and consequences. Adv Nutr.

[13] Thurnham DI, Davies JA, Crump BJ, Situnayake RD, Davis M (1986). The use of different lipids to express serum tocopherol: lipid ratios for the measurement of vitamin E status. Ann Clin Biochem.

[14] Gunanti IR, Marks GC, Al-Mamun A, Long KZ (2014). Low serum concentrations of carotenoids and vitamin E are associated with high adiposity in Mexican-American children. J Nutr.

[15] Wang JQ, Lin X, Bloomgarden ZT, Ning G (2020). The Jiangnan diet, a healthy diet pattern for Chinese. J Diabetes.

[16] Zaaboul F, Liu YF (2022). Vitamin E in foodstuff: nutritional, analytical, and food technology aspects. Com Rev Food Saf.

[17] Lwanga SK, Lamshhow S (1991). Sample determination in health studies; a practical manual.

[18] Péter S, Eggersdorfer M, Weber P, Weber P, Birringer M, Blumberg JB, Eggersdorfer M, Frank J (2019). Vitamin E intake and serum levels in the general population: a global perspective. Vitamin E in human health.

[19] Rosada A, Kassner U, Weidemann F, König M, Buchmann N, Steinhagen-Thiessen E (2020). Hyperlipidemias in elderly patients: results from the berlin aging study II (BASEII), a cross-sectional study. Lipids Health Dis.

[20] Lu JP, Lu Y, Wang XC, Li XY, Linderman GC, Wu CQ (2017). Prevalence, awareness, treatment, and control of hypertension in China: data from 1·7 million adults in a population-based screening study (China PEACE million persons project). Lancet.

[21] Kitsantas P, Wu H (2013). Body mass index, smoking, age and cancer mortality among women: a classification tree analysis. J Obstet Gynaecol Res.

[22] Péter S, Friedel A, Roos FF, Wyss A, Eggersdorfer M, Hoffmann K (2015). A systematic review of global alpha-tocopherol status as assessed by nutritional intake levels and blood serum concentrations. Int J Vitam Nutr Res.

[23] Rigotti A (2007). Absorption, transport, and tissue delivery of vitamin E. Mol Aspects Med.

[24] Kayden HJ, Traber MG (1993). Absorption, lipoprotein transport, and regulation of plasma concentrations of vitamin E in humans. J Lipid Res.

[25] Traber MG, Jialal I (2000). Measurement of lipid-soluble vitamins–further adjustment needed?. Lancet.

[26] Gouado I, Ejoh RA, Kenne M, Ndifor F, Mbiapo FT (2005). Serum concentration of vitamins A and E and lipid in a rural population of north Cameroon. Ann Nutr Metab.

[27] Sun GZ, Li Z, Guo L, Zhou Y, Yang HM, Sun YX (2014). High prevalence of dyslipidemia and associated risk factors among rural Chinese adults. Lipids Health Dis.

[28] Qi L, Ding X, Tang W, Li Q, Mao D, Wang Y (2015). Prevalence and risk factors associated with dyslipidemia in Chongqing, China. Int J Environ Res Public Health.

[29] Yao XG, Frommlet F, Zhou L, Zu F, Wang HM, Yan ZT (2010). The prevalence of hypertension, obesity and dyslipidemia in individuals of over 30 years of age belonging to minorities from the pasture area of Xinjiang. BMC Public Health.

[30] Mendes R, Themudo Barata JL (2008). Aging and blood pressure. Acta Med Port.

[31] Gross M, Yu X, Hannan P, Prouty C, Jacobs DR (2003). Lipid standardization of serum fat-soluble antioxidant concentrations: the YALTA study. Am J Clin Nutr.

[32] Khoo KL, Tan H, Liew YM, Sambhi JS, Aljafri AM, Hatijah A (2000). Blood pressure, body mass index, heart rate and levels of blood cholesterol and glucose of volunteers during national heart weeks, 1995–1997. Med J Malaysia.

[33] Liao CC, Su TC, Chien KL, Wang JK, Chiang CC, Lin CC (2009). Elevated blood pressure, obesity, and hyperlipidemia. J Pediatr.

[34] Oldewage-Theron WH, Samuel FO, Djoulde RD (2010). Serum concentration and dietary intake of vitamins A and E in low-income South. Clin Nutr.

[35] Assantachai P, Lekhakula S (2007). Epidemiological survey of vitamin deficiencies in older Thai adults: implications for national policy planning. Public Health Nutr.

[36] Ford ES, Schleicher RL, Mokdad AH, Ajani UA, Liu S (2006). Distribution of serum concentrations of alpha-tocopherol and gamma-tocopherol in the US population. Am J Clin Nutr.

[37] Obeid OA, Al-Ghali RM, Khogali M, Hwalla N (2006). Vitamins A and E status in an urban Lebanese population: a case study at Dar al-fatwa area, Beirut. Int J Vitam Nutr Res.

[38] Kang MJ, Lin YC, Yeh WH, Pan WH (2004). Vitamin E status and its dietary determinants in Taiwanese–results of the nutrition and health survey in Taiwan 1993–1996. Eur J Nutr.

[39] Sauberlich HE, Dowdy RP, Skala JH (1973). Laboratory tests for the assessment of nutritional status. Crit Rev Clin Lab Sci.

[40] Food and Agriculture Organization of the United Nations. Agriculture food and nutrition for Africa-A resource book for teachers of agriculture, http://www.fao.org/3/W0078E/W0078E00.html.; 1997. Accessed 21 Jan 2021.

