# Supplementary material for: The low prevalence rate of vitamin E deficiency in urban adults of Wuhan from central China: findings from a single-center, cross-sectional study
Source: Eur J Med Res. 2023 Mar 30;28:141. doi: 10.1186/s40001-023-01103-9 (PMC10062001; doi:10.1186/s40001-023-01103-9)
Supplement: Supplementary file 1 — Additional file 1: Table S1 Method validation. Table S2 Classification of the subjects according to their TC levels in adults (n=846). Table S3 Classification of the subjects according to their blood pressures in adults (n=846). Table S4 Classification of the subjects according to their BMI in adults (n=846). [file 40001_2023_1103_MOESM1_ESM.docx]

**The low** **prevalence rate of vitamin E deficiency in urban adults of Wuhan from central China: findings from a single-center, cross-sectional study**

**Ying Shen, Ke Liu, Xia Luo, Liming Cheng***

Department of Laboratory Medicine, Tongji Hospital, Tongji Medical College, Huazhong University of Science and Technology, Wuhan, 430030, China.

*** Correspondence:**

**Liming Cheng**, MD, Department of Laboratory Medicine, Tongji Hospital, Tongji Medical College, Huazhong University of Science and Technology. Wuhan 430030, PR China. Tel: 86-27-836-65468; E-mail address: [chengliming2015@163.com](mailto:chengliming2015@163.com).

**Table S1 Method validation**

| **Linearity** | Linearity of the method was established from 0.23-46 μmol/L by plotting the peak area ratio (analyte/IS) by least square linear regression analysis with a weighting factor of 1/x^2^. | | | | | | | | | |
| --- | --- | --- | --- | --- | --- | --- | --- | --- | --- | --- |
|  | Linear equation | | | | | linear correlation coefficient (r^2^) | | | Linear range  (μmol/L) | |
|  | y=0.358x+0.011 | | | | | 0.9987 | | | 0.232-46.4 | |
| **Precision** | Three levels of quality controls (1.2, 4.8, 24 μmol/L) were assayed five replicates on each day over five days to evaluate the intra-day and inter-day precision. Precision within 15 % was accepted. | | | | | | | | | |
|  | Intra-day precision (CV) | | | | | Inter-day precision (CV) | | | | |
|  | QCL | QCM | | QCH | | QCL | | QCM | | QCH |
|  | 4.91% | 3.73% | | 3.26% | | 5.04% | | 6.47% | | 4.46% |
| **Accuracy** | Accuracy was evaluated by the recovery experiment. Samples were spiked with a known amount of vitamin E at three different levels (1.2, 4.8, 24 μmol/L). All measurements were performed in five times and the recovery was calculated as [(final concentration-initial concentration)/added concentration]. Recovery within 85-115% was accepted. | | | | | | | | | |
|  | Level 1 | | | Level 2 | | | | Level 3 | | |
|  | 105.58% | | | 99.37% | | | | 101.02% | | |
| **Analytical**  **sensitivity** | Analytical sensitivity, expressed as the limit of quantification (LOQ), were defined as the analyte peak with a signal-to-noise of 10, and was established based on the criteria of maintaining the trueness within 80-120% and the variable coefficient (CV) <20%. | | | | | | | | | |
|  | Expected concentration (μmol/L) | Actual concentration (μmol/L) | | | | | | CV | | Trueness |
|  | 0.23 | 0.23 | 0.23 | 0.21 | 0.21 | 0.21 | 0.23 | 4.36% | | 95.6% |
| **Matrix effect** | Matrix effect was evaluated by the signal-recovery spiking experiment. The analyte added into the serum specimen and the pure solvent at three different levels (4.6, 11.5 and 23 μmol/L). The absolute matrix factor (MF) of the analyte and IS was calculated as Ai/Ai’ × 100% (where Ai and Ai’ represent the peak area of the analyte or IS within and without the matrix, respectively), and the relative MF of the analyte and IS are calculated as (MF of analyte)/ (MF of IS) × 100%. A relative MF within 85-115% was accepted. | | | | | | | | | |
|  | Level 1 | | | Level 2 | | | | Level 3 | | |
|  | 102 | | | 111 | | | | 112 | | |

**Table S2** Classification of the subjects according to their TC levels in adults (n=846)

|  | Hypercholesterolemia  (total cholesterol ≥ 5.2 mmol/L) | Normal  (total cholesterol < 5.2 mmol/L) | *P* |
| --- | --- | --- | --- |
| N (%) | 155 (18.32%) | 691 (81.68%) |  |
| Age (years) | 51 (43-57) | 45 (34-56) | <0.001 |
| BMI (kg/m^2^) | 24.5 (22.4-26.7) | 24.1 (21.7-26.1) | 0.081 |
| SBP (mm Hg) | 126 (116-142) | 123 (112-136) | 0.036 |
| DBP (mm Hg) | 76 (70-86) | 76 (68-85) | 0.325 |
| TC (mmol/L) | 5.68 (5.40-6.01) | 4.23 (3.79-4.66) | <0.001 |
| TG (mmol/L) | 1.55 (1.10-2.56) | 1.10 (0.78-1.59) | <0.001 |
| HDLC (mmol/L) | 1.31 (1.13-1.56) | 1.24 (1.06-1.47) | 0.007 |
| LDLC (mmol/L) | 3.83 (3.55-4.13) | 2.62 (2.20-3.04) | <0.001 |
| TLs (mmol/L) | 7.32 (6.74-8.27) | 5.42 (4.78-6.06) | <0.001 |

**Table S3** Classification of the subjects according to their blood pressures in adults (n=846)

|  | High blood pressure (≥140/90 mmHg ) | Normal  (<140/90 mmHg) | *P* |
| --- | --- | --- | --- |
| N (%) | 210 (24.82%) | 636 (75.18%) |  |
| Age (years) | 56 (46-70) | 44 (33-53) | <0.001 |
| BMI (kg/m^2^) | 25.3 (23.7-27.8) | 23.6 (21.3-25.7) | <0.001 |
| SBP (mm Hg) | 147 (142-158) | 118 (109-127) | <0.001 |
| DBP (mm Hg) | 91 (85-97) | 73 (66-80) | <0.001 |
| TC (mmol/L) | 4.52 (4.06-5.15) | 4.36 (3.85-4.97) | 0.005 |
| TG (mmol/L) | 1.54 (1.07-2.18) | 1.08 (0.78-1.56) | <0.001 |
| HDLC (mmol/L) | 1.15 (1.01-1.35) | 1.28 (1.09-1.51) | <0.001 |
| LDLC (mmol/L) | 2.85 (2.44-3.41) | 2.76 (2.25-3.30) | 0.058 |
| TLs (mmol/L) | 6.14 (5.42-7.13) | 5.52 (4.85-6.44) | <0.001 |

**Table S4** Classification of the subjects according to their BMI in adults (n=846)

|  | <18.5  (under weight) | 18.5-24.9  (normal weight) | 25.0-29.9  (over weight) | ≥ 30.0 (obese) | *P* |
| --- | --- | --- | --- | --- | --- |
| N (%) | 23 (2.72%) | 488 (57.68%) | 303 (35.82%) | 32 (3.78%) |  |
| Age (years) | 27 (21-29) | 47 (35-56) | 49 (40-57) | 41 (32-62) | <0.001 |
| BMI (kg/m^2^) | 17.9 (17.3-18.2) | 22.5 (21.0-23.9) | 26.5 (25.6-27.8) | 30.8 (30.3-33.0) | <0.001 |
| SBP (mm Hg) | 107 (99-121) | 119 (110-131) | 130 (119-142) | 139 (128-147) | 0.001 |
| DBP (mm Hg) | 68 (61-75) | 75 (66-82) | 81 (74-88) | 83 (71-98) | 0.008 |
| TC (mmol/L) | 3.88 (3.57-4.08) | 4.40 (3.84-4.96) | 4.47 (3.99-5.11) | 4.60 (3.93-5.02) | 0.001 |
| TG (mmol/L) | 0.70 (0.54-0.89) | 1.00 (0.72-1.45) | 1.47 (1.08-2.17) | 1.62 (1.34-2.06) | <0.001 |
| HDLC (mmol/L) | 1.63 (1.45-1.76) | 1.33 (1.14-1.57) | 1.15 (1.00-1.32) | 1.10 (0.88-1.22) | <0.001 |
| LDLC (mmol/L) | 2.18 (1.84-2.39) | 2.75 (2.26-3.26) | 2.85 (2.43-3.50) | 3.15 (2.48-3.47) | <0.001 |
| TLs (mmol/L) | 4.60 (4.36-4.93) | 5.50 (4.81-6.32) | 6.06 (5.28-7.15) | 6.17 (5.46-7.06) | <0.001 |
